# Tire Rubber-Derived Cyclic Amines in Urban Ambient Particulate Matter in Shanghai

**DOI:** 10.1021/acsearthspacechem.4c00291

**Published:** 2025-03-20

**Authors:** Munila Abudumutailifu, Chengze Li, Haiping Xiong, Sihan Liu, Chunlin Li, Dongmei Cai, Yinon Rudich, Jianmin Chen

**Affiliations:** † Shanghai Key Laboratory of Atmospheric Particle Pollution and Prevention (LAP3), National Observations and Research Station for Wetland Ecosystems of the Yangtze Estuary, Department of Environmental Science & Engineering, 12478 Fudan University, Shanghai 200438, China; ⊥ IRDR International Center of Excellence on Risk Interconnectivity and Governance on Weather/Climate Extremes Impact and Public Health, Institute of Atmospheric Sciences, Fudan University, Shanghai 200438, China; ‡ College of Environmental Science and Engineering, Tongji University, Shanghai 200072, China; ∥ Department of Earth and Planetary Sciences, 34976 Weizmann Institute of Science, Rehovot 76100, Israel

**Keywords:** tire wear chemicals, PM_2.5_, cyclic amine, VACES, health risks

## Abstract

Tire wear compounds (TWCs) and their byproducts have raised increasing environmental and health concerns due to their widespread production and release. In this study, a custom-designed versatile aerosol concentration enrichment system coupled with HPLC-Q-TOF-MS was employed to conduct a nontargeted screening of suspect TWCs in urban PM_2.5_, followed by the targeted quantification of ten selected TWCs, providing high temporal resolution data across summer, autumn, and winter in Shanghai. The total TWC concentrations (∑TWCs) exhibited distinct seasonal variations. The highest levels were observed in autumn, with an average concentration of 15.53 ng/m^3^ (ranging from 1.39 to 58.67 ng/m^3^), followed by summer with an average of 7.44 ng/m^3^ (2.22 to 25.39 ng/m^3^), and the lowest levels observed in winter, with an average of 5.74 ng/m^3^ (1.56 to 17.83 ng/m^3^). The seasonal contributions of ∑TWCs were 73.9% in the autumn, 18.6% in the summer, and 7.5% in the winter. The diurnal pattern showed elevated nighttime concentrations compared with morning and evening rush hours. This study marks the first to investigate the diurnal variation in the ratio of *N*-(1,3-dimethylbutyl)-*N*′-phenyl-*p*-phenylenediamine quinone (6PPD-Q) to its parent compound, *N*-(1,3-dimethylbutyl)-N′-phenyl-*p*-phenylenediamine (6PPD), in the atmospheric particle phase. The ratio showed a similar daily pattern, peaking in the afternoon and reaching 3.64 in the summer and 6.55 in the autumn, in alignment with temperature and ozone patterns. Correlation analysis showed weak relationships between ∑TWCs and temperature (*R* = 0.12) as well as a weak negative correlation with humidity (*R* = −0.04). These findings highlight the need for further research into the toxicological and epidemiological impacts of TWCs, especially considering the heightened levels of nighttime exposure among night workers.

## Graphical abstract

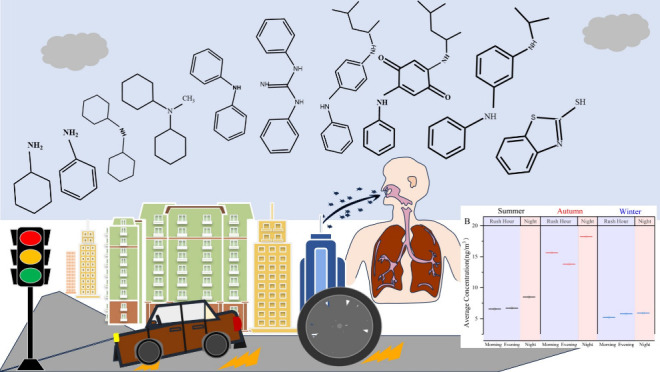

## Introduction

Pollution from car tires has become an increasing concern due to its significant contribution to nonexhaust emissions in urban environments and its potentially toxic effects.^1^ Nonexhaust emissions, including particulate matter (PM) from brake and tire wear, road surface abrasion, railway friction, and the resuspension of road dust, can account for up to 90% of traffic-related PM by mass and significantly elevate PM_10_ and PM_2.5_ levels, thereby exacerbating urban air pollution.^2,3^ Among these sources, brake dust constitutes 55.3 ± 7.0% and tire dust 10.7 ± 2.3% of the nonexhaust particle mass.^4^ The recycling of end-of-life tires into crumb rubber for synthetic turf further raises environmental concerns due to the continuous emission of substances related to tire emissions.^5^ At the same time, the growing adoption of electric vehicles, driven by their zero tailpipe emissions, has introduced new challenges. These vehicles are approximately 24% heavier than conventional cars of the same type, resulting in increased tire wear emissions.^3^ Tire wear generates complex mixtures known as tire wear compounds (TWCs), which are significant contributors to urban PM_2.5_ and pose serious environmental and health risks.^6–10^

Studies indicate that these surfaces release pollutants, exacerbated by factors such as high temperatures, UV exposure, and ozone, which may accelerate the breakdown of tire-derived compounds.^11^ These compounds, identified as significant contributors to the oxidative potential of PM_2.5_, have been found to increase significantly alongside roads.^7,12–14^ The latest study discusses a potential competitive mechanism between the formation of 6PPD-quinone and secondary nitrosamine (SNA).^6^ These substances can enter the human body via inhalation and ingestion. Growing evidence suggests that tire particles can penetrate human and animal bodies through air, soil, and water pathways.

A study employed two-dimensional gas chromatography equipment coupled with a time-of-flight mass spectrometer (GCxGC-TOF-MS) to analyze compounds released from heated tire samples (see https://www.emissionsanalytics.com/tyre-emissions) and showed that nitrogen-containing compounds are frequently identified as carcinogens, demanding urgent attention. Many cyclic amines, including various diphenylamine antioxidants (PPD-Q), have been reported in the literature for their association with tire wear. Jiang et al.^15^ identified 81 substances in the water-leachable fraction of tire wear particles (TWPs), with cyclic amines (CA) accounting for 46% and exhibiting toxicity to S. obliquus. A recent study detected diphenylamine (DPA), 1,3-diphenylguanidine (DPG), and *N*-phenyl-*N*′-(1,3-dimethylbutyl)-*p*-phenylenediamine (6PPD) in road dust, with detection frequencies (DF, percent) exceeding 70%.^16^ They were found in both PM_2.5_ and water discharge from the tire production plants.^17–19^ *N*-(1,3-Dimethylbutyl)-*N*′-phenyl-*p*-phenylenediamine quinone (6PPD-Q), DPG, *N*-methyl-dicyclohexylamine (DCA), 1,3-dicyclohexylurea­(DCU), and 1-cyclohexyl-3-phenylurea (CPU) were detected in urban runoff and correlated well with roads and residential land-use areas.^20^ Aniline (ANI), cyclohexylamine (CHA), DPA, and 6PPD-Q were detected from cryogenically milled tire tread (CMTT) extracts as well as simulated gastrointestinal extracts.^21^ Health risks and the possibility of maternal transfer have been reported for DPG, dicyclohexylamine (DChA), 6PPD­(-Q), and DPA,^22^ while inhalation has been identified as the main route of human exposure to atmospheric microplastics including TWPs, accounting for over 85% of the total exposure in most cases of this study.^23^ A study suggested that a number of chlorinated products of 6PPD-Q from disinfection enhance the toxicity of 6PPD-Q toward zebrafish embryos.^24^ Recent findings of 6PPD-Q in human samples, including blood,^25^ urine,^26^ and cerebrospinal fluid, highlight the urgent need for additional research into the public health and toxicological impacts of these compounds.

The objectives of this study were (1) to use both nontarget and target methods to identify and quantify tire-wear-related compounds (especially cyclic amines) in PM_2.5_ samples; (2) to investigate diurnal variations across different seasons in Shanghai; and (3) to examine the relationship between atmospheric environmental factors and meteorological conditions related to TWCs. For the first time, the concentrations of TWCs were investigated with high temporal resolution including the newly discovered transformation product 6PPD-quinone in atmospheric fine particulate matter. These findings further underscore the urgent need for comprehensive research on the impact of tire wear emissions on air quality and human health.

## Methods

### Sample Collection

The PM_2.5_ samples were collected using a custom-designed versatile aerosol concentration enrichment system (VACES), with detailed specifications provided in our previous research.^27–30^ Briefly, particles underwent supersaturation, condensation, and particle acceleration (to separate gases and particles) before being introduced into a low-volume automatic sampling system (Comdederenda, China) for PM_2.5_ sample collection, which can concentrate ambient aerosols by up to 10-fold. The sampling campaign was conducted at Fudan University in Shanghai during three seasons: June (summer) and October (autumn) of 2021, as well as January (winter) of 2022, respectively (Figure S1). The sampling site was situated on the seventh floor of the John Ling Building at the Jiangwan Campus of Fudan University (31.344° N, 121.518° E). PM_2.5_ samples were collected on 47 mm-diameter prebaked quartz-fiber filters (Whatman Company, Leicestershire, U.K.). For details on ambient PM_2.5_ concentrations during the sampling period, please refer to Figure S1. All collected samples were labeled and stored at −20 °C in a freezer until pretreatment and instrumental analysis.

### Extraction and Chemicals

The membrane samples were extracted with Methanol (MeOH). To ensure the thoroughness of the MeOH ultrasound extraction, three consecutive ultrasonographic extractions were performed. To prevent cross-contamination, the tweezers, ceramic scissors, and needles (for blowing nitrogen gas) were cleaned between samples using methanol and ultrapure water and dried. Complete details of the extraction protocols are found in the Supporting Information, Text S1. HPLC-grade solvents, including water and methanol, were purchased from J.T.Baker. Formic acid (FA) was purchased from Fisher Scientific (Ottawa, ON, Canada). Ten standards including cyclohexylamine (CHA), dicyclohexylamine (DCHA), *N*-methyl-dicyclohexylamine (DCA), aniline, diphenylamine (DPA), *N*,*N*′-diphenylguanidine (DPG), *N*-(1,3-dimethylbutyl)-*N*-phenyl-*p*-phenylenediamine (6PPD), 2-anilo-5-cyclohexa-2,5-diene-1,4-dione (6PPD-Q), *N*-isopropyl-*N*-phenyl-1,4-phenylenediamine (IPPD), and 2-mercaptobenzothiazole (MBT) were purchased. Quinoline-*d*_7_ was acquired from First Standard (Altascientific, china) and used as an internal standard. See Tables S1 and S2 for complete chemical reagent details and properties.

### UHPLC-Q-TOF-MS Analysis

Analysis was conducted using a UHPLC 1290 series system coupled to a quadrupole time-of-flight (Q-ToF) mass spectrometer series 6540 (Agilent Technologies, Santa Clara, CA) at a high resolution (2 GHz). LC separation was performed using a ZORBAX Eclipse Plus C18 column (1.8 μm, 2.1 × 50 mm; Agilent Technologies) by gradient elution with LCMS grade water and methanol (J.T.Baker), both containing 0.1% formic acid (FA) at a flow rate of 0.35 mL min^–1^ and a column temperature of 40 °C. For LC separate parameters, see Table S3.

## Results and Discussion

### Identification of Tire-Related Cyclic Amine

Suspect screening approaches optimized for liquid chromatography and gas chromatography, coupled with high-resolution mass spectrometry (LC- or GC-HRMS), have been effectively employed to identify newly emerging compounds of environmental or health concern.^31–33^ Most PPD-related compounds, including PPD-quinones (PPD-Q), are derived from the natural oxidation of PPDs and are difficult to obtain as commercial standards, thus requiring self-synthesis. Suspect compounds were tentatively identified following the approach outlined by Gago-Ferrero et al.^32^ Criteria for feature selection included (1) peak area (≥200 for ESI (+) and ≥100 for ESI (−)), intensity counts (≥100 for ESI (+) and ≥50 for ESI (−)); (2) mass accuracy (±2 mDa/5 ppm); (3) isotopic pattern match; (4) the peak score; (5) retention time consistency with a quantitative structure retention relationship model (QSRR); (6) the presence of characteristic adducts; (7) MS/MS spectral interpretation, performed using spectral libraries (MassBank database https://massbank.eu/MassBank/) and expert input. The framework for screening suspect compounds was proposed by Schymanski et al.,^34^ ranging from confirmed structures (level 1) to exact masses of interest (level 5), with intermediate levels based on spectrum matches, diagnostic evidence, and molecular formulas. Nontarget analysis was conducted using the molecular features algorithm in MassHunter10.0 (Agilent). The molecular formula(s) for the selected peaks were assigned based on the parameters used in steps (1), (2), (4), and (6) outlined earlier. A peak score of >85 was required at this stage. The MS/MS spectral interpretation was conducted using both the MassBank library and the literature references. Based on previous studies,^16,35–39^ a set of diagnostic fragment ions previously reported for PPD-related compounds—*m*/*z* 184.09, 107.06, 167.07, 168.07, 169.08, and 93.05—was used to broadly identify potential candidates. Additionally, Kendrick mass defect (KMD) values from known standards (6PPD and 6PPD-Q) were used to support identifying related compounds within homologous series.^40^ Once candidates were identified, confirmation was achieved by integrating specific diagnostic fragment ions and verifying consistency with the expected fragmentation patterns.

A suspect screening list was established comprising PPD antioxidants and their potential quinone derivatives (Figure 1). We detected *m*/*z* 297.2336, with the molecular formula C_20_H_28_N_2_, matched by the Agilent MassHunter Workflows, along with fragment ions *m*/*z* 184.1025, 107.0613, and 168.0702 at 16.5 min (Figure S3), sharing the same KMD-CH_2_ with 6PPD, and reasonably speculated to be 8PPD. Similarly, *m*/*z* 325.2656, (C_22_H_32_N_2_) at 25.7 min, displayed a similar KMD (0.105), indicating that it belonged to the same homologous series as 6PPD and 8PPD. Additional compounds, including C_14_H_24_N_2_ (44PD, *m*/*z* 221.2045) and C_12_H_20_N_2_ (33PD, *m*/*z* 193.1614), were confirmed using specific fragment ions and consistent KMD values (0.05). For example, at 13.7 min, we observed the *m*/*z* 221.2045 with the best fragment ions m/z107.0692, 169.0851 corresponding to formula C_14_H_24_N_2_ (44PD). At 6.72 min, we observed *m*/*z* 193.1614, which matched C_12_H_20_N_2_, with fragment ions *m*/*z* 107.0784 and *m*/*z* 167.0659 *m*/*z* 168.0785. At 24.2 min, *m*/*z* 273.2383 was observed, which matched C_18_H_28_N_2_ and was tentatively assigned to CCPD. Among these compounds, research indicates that 6PPD, CPPD, and 44PPD were found in human urine.^36^

**Figure 1:**
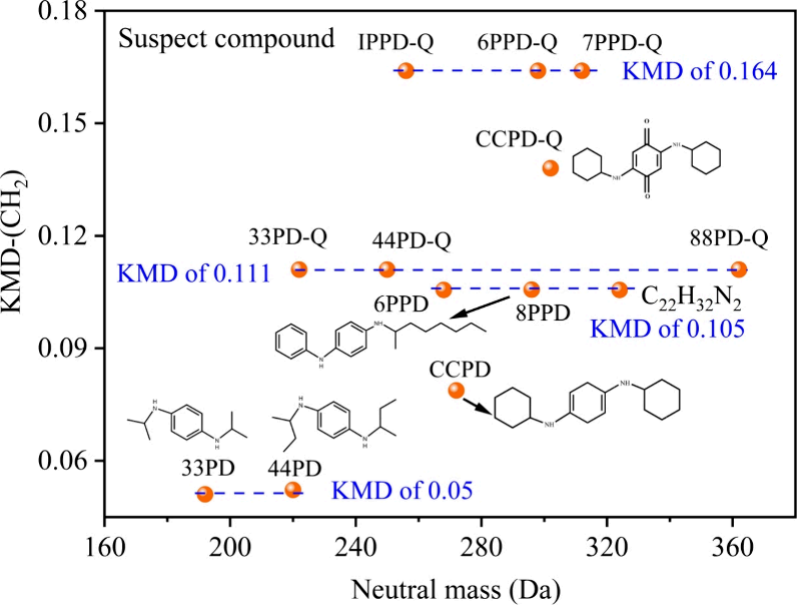
Kendrick mass defect diagram of PPD and PPD-quinones. Consistencies in the KMD of suspect molecules are marked with blue dash line.

For the MS/MS spectral interpretation of PPD-Q, we adopted the methodology outlined by Wang et al.,^41^ who categorized known PPD-quinones into three main classes. Class I (IPPD-Q, CPPD-Q, and 6PPD-Q) was defined by characteristic fragment ions such as *m*/*z* 215.0815 (C_12_H_11_N_2_O_2_^+^), 187.0866 (C_11_H_11_N_2_O^+^), and 170.0600 (a stable five-membered ring structure). Additional PPD-quinones, classified as classes II and III, were identified using supplementary fragment ions (*m*/*z* 170.0600, 184.0757, and 139.0502), which revealed structural features such as carbonyl and aniline groups,^41^ along with characteristic neutral losses (e.g., 199.0633 and 138.0429). Based on this fragmentation, we observed *m*/*z* 363.3003 at 25.07 min, with fragment ions *m*/*z* 187.0830, 215.0867, and 199.0635. This spectrum matched the molecular formula C_22_H_38_N_2_O_2_, and the compound was tentatively assigned as 88PPDQ. Similarly, at 11.61 min, *m*/*z* 251.1749 was observed, with fragment ions *m*/*z* 187.0730, 215.0851, and 169.0524, consistent with the molecular formula C_14_H_22_N_2_O_2_, and likely corresponding to 44PD-Q (Figure S4). In addition, at 6.05 min, we observed the *m*/*z* 223.1435, which matched 33PPD-Q, and shared the same KMD values (e.g., 0.111) with 44PPD-Q and 88PPD-Q. Furthermore, IPPD-Q and 7PPD-Q were found to have the same KMD value (0.164) as 6PPD-Q. At 20.18 min, *m*/*z* 303.2065 was observed, matching C_18_H_26_N_2_O_2_ and tentatively assigned to CCPD-Q.

Overall, suspect compounds and their respective confidence levels are summarized in Table S4. Some suspect compounds, such as CCPD and CCPD-Q, lacked direct diagnostic fragment ions and were identified solely based on molecular formulas and precursor ion information. These compounds were classified at confidence level 4 due to the absence of direct MS/MS evidence.

### Concentrations and Seasonal Contribution of Target TWCs

Ten target TWCs were found in the samples (Table 1) and confirmed by standards (Table S1). Among these, nine tire-related cyclic amines associated with tire emissions were detected in Shanghai from 2021 to 2022 (Figure S1), including CHA, DCHA, DCA, aniline, DPA, DPG, 6PPD, IPPD, and 6PPD-Q. Their structures are summarized in Figure S5. Each compound has been identified in various studies as a pollutant related to tire emissions and extensively characterized due to its presence in environmental samples.^1^ Table 2 presents quantitative data on these TWCs in PM_2.5_ samples across different seasons, showing varying detection frequencies (DF) in atmospheric particulates.

**Table 1:** Full Names, Abbreviations, Precursor and Product Ion *m*/*z* of Target Tire Wear Compounds (TWCs)

full name	abbreviations	retention time (min)	precursor ion (*m*/*z*)	product ion (*m*/*z*)
cyclamine	CHA	1.19	100.1124	83.08; 55.05; 68.98; 72.63
dicyclohexylamine	DCHA	4.90	182.1911	136.99; 100.17; 83.09; 55.06
1-cyclohexyl-3-phenylurea	DCA	4.71	196.2509	114.1308; 83.0949; 55.0610
aniline	ANI	0.73	94.0658	77.0386; 51.0230; 67.0541
diphenylamine	DPA	14.39	170.0966	152.06; 93.06; 77.04; 65.04
1,3-diphenylguanidine	DPG	3.90	212.1814	195.09; 119.06; 94.06; 77.04
4-(isopropamino)diphenamine	IPPD	7.54	227.1540	184.09; 107.06; 93.05
*N*-phenyl-*N*′-(1, 3-dimethylbutyl)-*p*-phenylenediamine	6PPD	12.46	269.2017	184.1031; 107.0622; 93.0605
*N*-(1,3-dimethylbutyl)-*N*′-phenyl-*p*-phenylenediamine quinone	6PPD-Q	18.54	299.1775	256.11; 241.09; 215.08; 187.08
mercaptobenzothiazole	MBT	12.4	167.9936	124.02; 109.01; 133.07

**Table 2:** Descriptive Statistics for Concentrations (pg/m^3^, (Range, Average)) and Detection Frequencies (DF, %) of TWCs in PM_2.5_ from Cities in Shanghai across the Different Seasons^a^

	summer (*N* = 100)			autumn(*N* = 95)			winter(*N* = 103)		
compound	range	average	DF	range	average	DF	range	average	DF
CHA	(0.1–17.4) × 10^3^	4.1 × 10^3^	100	(0.2–28.8) × 10^3^	101 × 10^3^	100	0.1–14.5 × 10^3^	3.4 × 10^3^	100
DCHA	(1.2–7.2) × 10^3^	2.5 × 10^3^	100	(0.01–29.8) × 10^3^	4.0 × 10^3^	100	(0.6–5.0) × 10^3^	1.7 × 10^3^	100
DCA	3.7–28.5	8.3	100	0.96–45.80	8.6	100	ND–6.3	4.0	98
Aniline	ND–288.6	76.1	80	0.9–423.2	65.1	100	ND–58.2	18.7	46
DPA	ND–209.1	51.5	88	(0.03–13.4) × 10^3^	398.9	100	5.8–97.3	55.4	100
DPG	167.0–318.5	192.9	80	60.2–331.1	126.0	100	ND–263.9	124.6	99
6PPD	ND–194.9	43.7	98	27.8–277.7	100.8	100	BLD–39.4	27.8	97
IPPD	ND–990.1	129.8	92	21.5–1129.4	243.7	100	5.5–839.4	233.7	100
6PPD-Q	ND–552.9	100.7	96	1.0–1296.5	355.2	100	ND	ND	0
MBT	ND–687.09	180.5	88	43.01–67.61	49.9	100	ND–412.06	216.4	96

a BDL= below detection limit; ND= not detected.

The concentrations of all TWCs (∑TWCs) varied among different seasons, ranging from 2.22 to 25.39 ng/m^3^ in the summer, 1.39 to 58.67 ng/m^3^ in the autumn, and 1.56 to 17.83 ng/m^3^ in the winter (Table 2). The PM_2.5_ concentrations exhibited a wide range across seasons (Figure S1), with average values of 21.21 μg/m^3^ in summer, 17.17 μg/m^3^ in autumn, and 47.56 μg/m^3^ in winter during the sampling period. The average contributions of target TWCs to PM_2.5_, as calculated in our study, were 0.05% in summer, 0.19% in autumn, and 0.02% in winter. Atmospheric particles with a large specific surface area can adsorb various organic and inorganic substances, including TWCs. These compounds are widely present in atmospheric particles and exhibit considerable temporal variability. To facilitate a precise comparison of the seasonal contributions of tire emissions to total particulate pollution, pollutant contributions were standardized by normalizing TWC concentrations with PM_2.5_ levels. In our study, a significant seasonal contribution was observed in TWC concentrations (Figure 2B), with the highest proportion in autumn (73.9% of the total average TWCs), followed by summer (18.6%), and unexpectedly, the lowest proportion occurred in winter (7.5%). Among the TWCs, CHA and DCHA were the most dominant contributors for all seasons. CHA, an impurity and byproduct in tire materials and tire wear particles,^42^ showed a DF of 100% in all seasons. This may be due to the fact that amine-based tire-wear-related compounds are prone to degradation or transformation, with cyclohexylamine (CHA) being one of the potential degradation products. The average concentration of CHA in autumn was 1149.35 pg/μg, accounting for 45.25% of the total CHA in the three seasons, which was significantly higher than in summer (267.02 pg/μg, contributing 10.51%) and winter (117.64 pg/μg, 4.63%). Lin et al.^43^ Conducted a study on emerging contaminants in PM_2.5_ sampled in Beijing and found a high concentration of CHA (not detected, 4.96 × 10^3^ pg/m^3^), suggesting the presence of identified Toxicity Forecaster (ToxCast) chemicals. DCHA was reported to dominate the chemical composition of recycled tire products and tire leachates^44^ and was detected in urban, agricultural watersheds, and sediment pore water after tire particle exposure.^45^ Despite the growing body of evidence linking DCHA to tires, atmospheric concentration data remain notably insufficient (Table S6). In our study, DCHA concentration was in the range of 11.8 pg/m^3^ to 29.7 ng/m^3^ in autumn, contributing 23.46% of total TWCs. The contribution is also much higher than summer (163.60 pg/μg, contributing 6.19%) and winter (49.16 pg/μg, contributing 1.86%). DPG is used in tire rubber as a secondary accelerator in silicon tread mixes (also called “activator”),^46^ and was previously shown to be released from tires.^45,47,48^ Notably, DCA is also identified within the Coho Mortality Signature, found in tire leachate, and also suggested as originating from tires.^48^ Comparatively, the DPG ranges 60.28–331.08 pg/m^3^ and contributes 0.55% in autumn, which is comparable with the level of summer DPG (167.04–318.51 pg/m^3^, contributing 0.45%) but higher than the level (ND–263.85 pg/m^3^, contributing 0.19%) in winter. As determined by its calculated logKOA, 94% of DPG could be distributed on the particle phase of atmospheric PM,^1^ is widely detected in indoor dust,^49^ and is generally detected in atmospheric mixtures in 18 major megacities.^50^ Aniline is used as a vulcanization accelerator and an antioxidant during rubber processing. A previous study showed that manufacturing rubber products for the automobile industry (three plants, all jobs combined) had a median aniline exposure of 2.5 μg/m^3^ (range, 1.0–37.4 μg/m^3^) in the breathing zone,^51^ and a relatively high concentration was found in ambient air (Table S6). A recent study conducted a study combining source apportionment with characteristic molecular markers indicated that TWPs contributed 13 ± 7% of urban PM_2.5_.^52^

**Figure 2:**
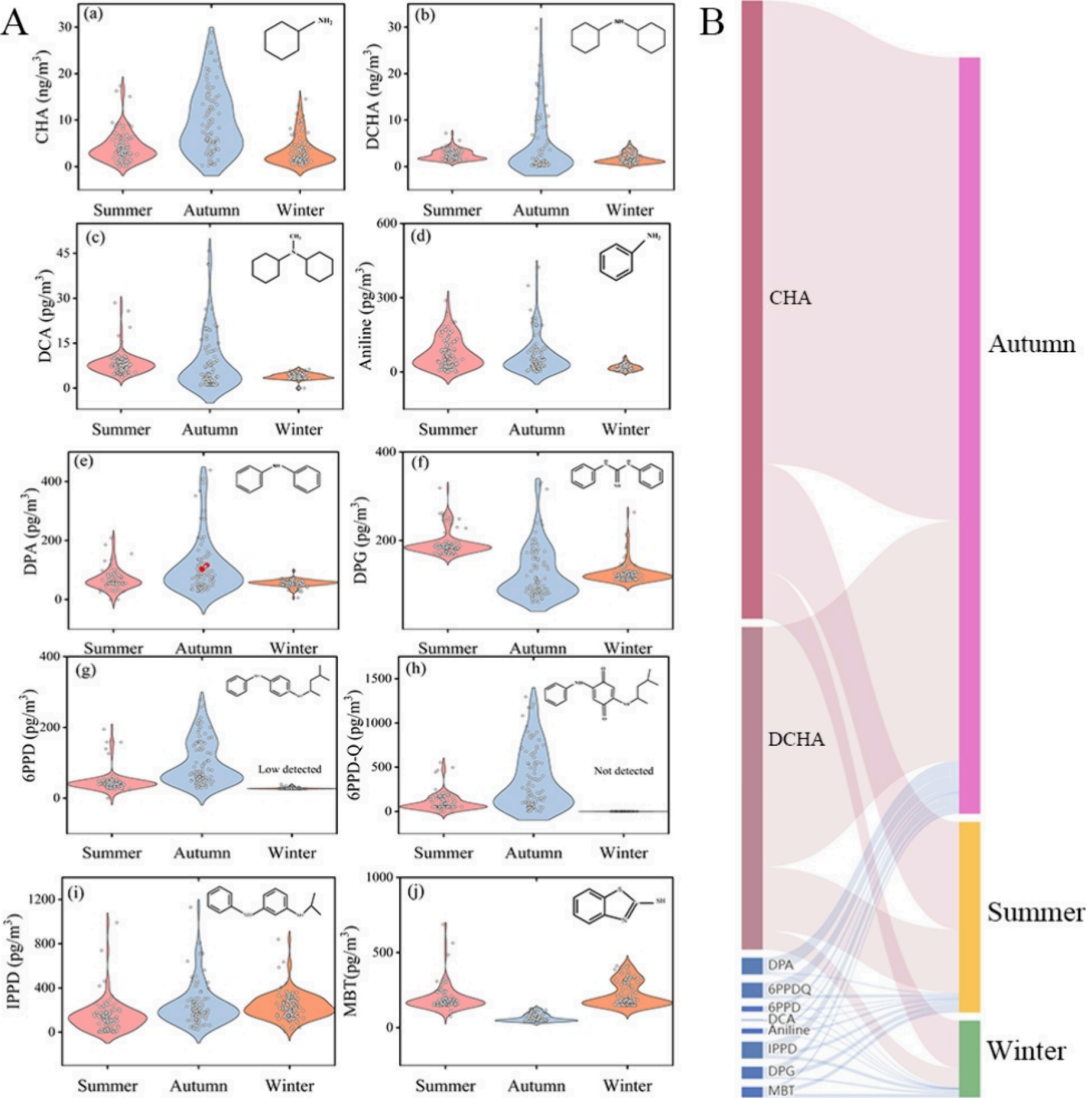
Violin plot of 10 target TWCs compounds (A). Concentrations of different substances represented in subfigures of panel (A): (a) CHA, (b) DCHA, (c) DCA, (d) aniline, (e) DPA, (f) DPG, (g) 6PPD, (h) 6PPD-Q, (i) IPPD, and (j) MBT. Two large DPA points were square-root transformed for better visualization and are shown as red dots. Mass fractions of TMCs were measured during different seasons over the entire sampling period (B).

In our study, the cumulative contribution of two types of PPD, and 6PPD-Q to PM_2.5_, was more than three times higher in autumn compared to summer, reaching up to 2.6 and 0.4% in winter. Currently, most of these antioxidants are believed to be released primarily from rubber abrasion. Tire wear particles, generated through tire friction on the road, have been confirmed as the major source of PPDs in the environment. In contrast, MBT showed the highest contribution in summer (0.40%) and the lowest in autumn (0.28%). The high detection frequencies are likely explained by the extensive use of these compounds.

In this study, the calculated target tire-wear pollutant concentrations showed distinct seasonal variations with the highest levels in autumn, intermediate levels in summer, and the lowest levels in winter. In winter, cooler temperatures lead to tire rubber hardening, reducing wear and particle emissions. In contrast, summer’s high temperatures may soften the tire rubber, but frequent rainfall during this season (RH = 100 contributing 34%) can result in reduced friction between tires and the road, subsequently decreasing particle emissions. Additionally, rainwater may wash away some tire particles, further lowering the measured concentrations. Moreover, summer tires tend to employ softer rubber compounds, which wear out more quality, while winter tires have wider channels between the “lugs”, which means that there is less rubber contact with the road surface.

Atmospheric conditions also influence the climate and air quality dynamics during these seasons. Autumn is often characterized by stagnant air, which can hinder the dispersion of pollutants. This stagnation results in a buildup of airborne particles, leading to higher concentrations of tire wear pollutants. In contrast, summer typically promotes better atmospheric mixing, aiding in the dilution and dispersal of pollutants and contributing to lower observed concentrations. Moreover, the dry conditions in autumn may prolong the residence time of tire wear particles in the atmosphere, exacerbating their concentration.

### Diurnal Variation of TWCs and 6PPdQ/6PPd Ratio

Figure 3A shows that the total concentrations of all TWCs (∑TWCs) do not exhibit a significant diurnal variation pattern. To explore the variation further, the day was divided into the morning rush, evening rush, and night periods for comparison. Figure 3B reveals that nighttime concentrations were relatively higher than those observed during the two rush-hour periods (morning and evening). In summer, the concentrations of TWCs at night was 8.49 ± 5.0 ng/m^3^, higher than during the evening rush hour (6.71 ± 3.04 ng/m^3^) and morning rush hour (6.56 ± 2.39 ng/m^3^). Similarly, in autumn, the concentration of TWCs at night was 18.25 ± 16.27 ng/m^3^, higher than in the morning rush hour at 15.65 ± 11.67 ng/m^3^, and evening rush hour at 13.80 ± 12.04 ng/m^3^. In winter, evening rush and nighttime concentrations were similar, averaging around 5.7 ng/m^3^, both higher than the morning rush hour concentration of 5.20 ± 2.47 ng/m^3^.

**Figure 3:**
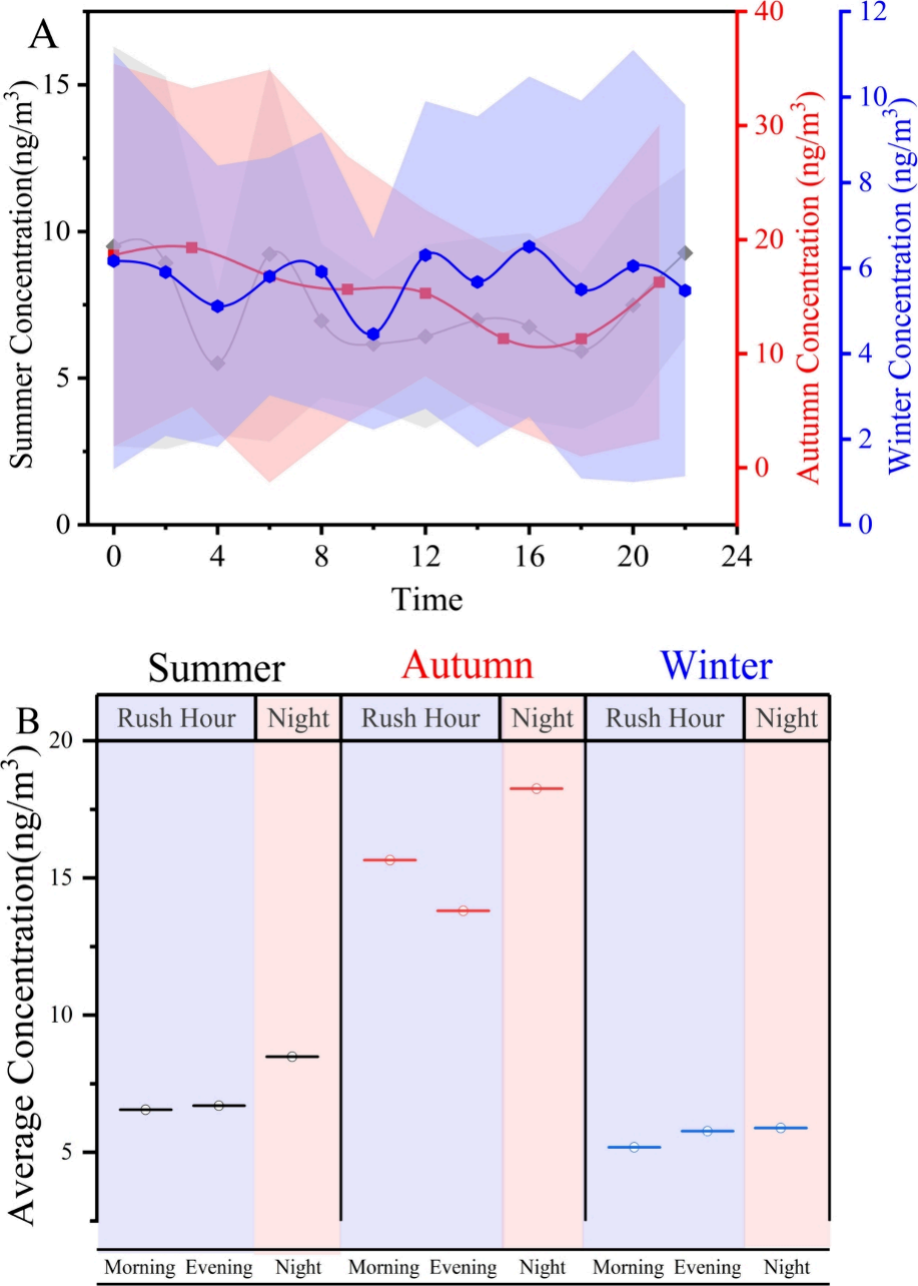
Diurnal variation of TWCs in different seasons (A). The black line represents the diurnal variation pattern in summer, the red line represents the pattern in autumn, and the blue line represents the pattern in winter. The shaded areas indicate the standard deviation. (B) Average concentration of TWCs during the morning, evening rush hours, and nighttime.

Due to the lack of studies with high time resolution, it is challenging to make comparative analyses. A recent study on the diurnal variation of TWCs, which involved relatively short-term sampling in a long tunnel in Xi’an,^19^ found that the highest concentration occurred during the evening rush hour (16:30–19:30), while the lowest concentration was during the morning rush hour (7:30–10:30). However, tunnels provide a confined space where pollutants can accumulate more easily, whereas the atmospheric environment is more complex compared to the confined tunnel environment. This discrepancy suggested that the abundance of TWCs was influenced not only by the number of vehicles, but also by meteorological variables, road washing activities, vehicle and tire types, and other unidentified factors.^19^ Higher nighttime concentrations may be attributed to stable atmospheric conditions, boundary layer dynamics, increased nighttime driving speeds, and other cumulative effects, resulting in the highest levels observed at night.

We observed that 6PPD-Q levels ranged from <LOQ to 1296 pg/m^3^, often exceeding 6PPD levels during most sampling times. This can be explained by the 6PPD-Q/6PPD ratio within each sample, where 97% of the summer samples and 71% of the autumn samples had a ratio greater than one. Figure 4 illustrates the diurnal variation of the 6PPD-Q/6PPD ratio, showing a similar daily trend in both summer and autumn. In autumn, the highest average ratio was 6.55, occurring between 12:00 and 15:00, while in summer, the highest ratio was 3.64, occurring between 12:00 and 14:00. This trend aligns with the daily variation patterns of the temperature and ozone concentration, suggesting that more attention should be paid to the oxidation process of PPD to PPD-Q.

**Figure 4:**
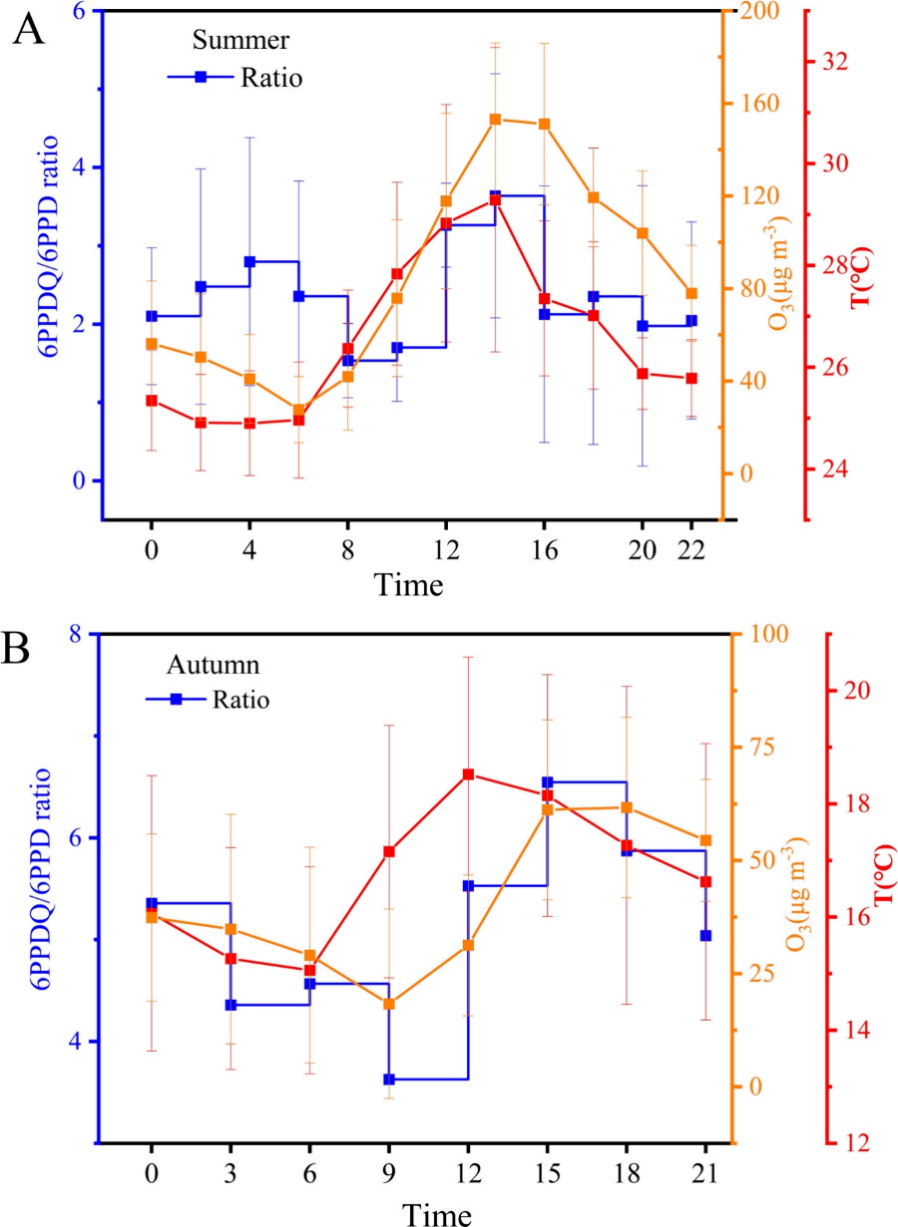
Diurnal variation of the 6PPD/6PPDQ ratio in summer (A) and autumn (B). The blue line represents the ratio of 6PPDQ/6PPD, the red line represents the diurnal variation of the temperature, and the orange line represents the diurnal variation of ozone.

Given the increasing concerns about environmental incidents caused by 6PPD-quinone, research on the environmental occurrence and risks of antioxidants and their transformation products (TPs) is growing. The formation and impacts of TPs resulting from photodegradation, thermal degradation, and biodegradation processes deserve further attention. Zhao et al. suggested that high temperatures significantly accelerate the decay of PPDs in rubber particles compared to UV exposure.^11^ Exposure to sunlight and high temperatures exacerbates the instability of TRWPs by promoting the conversion of 6PPD to 6PPD-Q through established scientific pathways, and studies have shown that sunlight exposure affects the number of extractable compounds more strongly than elevated temperatures, causing a clear shift from parent compounds to their TPs.^53^

### Effects of Meteorological Conditions on TWCs

Figure 5 shows the correlation analyses of temperature (*T*) and relative humidity (RH%) with tire wear emissions, where the correlation with temperature was *R* = 0.12 and weakly negatively correlated with humidity *R* = −0.04. This indicates a weak relationship between the temperature and tire wear emissions, while the influence of humidity appears even more negligible. In summer, except during rainy weather (RH = 100%), the correlations of *T* and RH were 0.05 and −0.16, respectively, suggesting that in summer, temperature exerts a minor positive effect on tire wear emissions, whereas humidity shows a slight negative impact. However, when examining the specific time periods in summer (Figure 5C,D), it becomes clear that in summer, between 14:00–16:00 and 16:00–18:00, the correlation between TWC concentrations and temperature peaked at 0.7 and 0.6, while there was a relatively strong negative correlation between TWC concentrations and RH during this time. This pattern suggests that during the hottest parts of the day in the summer, tire wear emissions increase significantly with temperature, while higher humidity levels might suppress the concentration of tire wear particles in the air. In winter, the temperature showed a significant correlation with TWC concentrations during all time periods except for 14:00–16:00, with relatively strong positive correlations. Interestingly, humidity was positively correlated with TWC concentrations during most time periods, except between 2:00 and 6:00 at night and during the morning rush hours (8:00–10:00) where humidity showed a negative correlation with TWCs.

**Figure 5:**
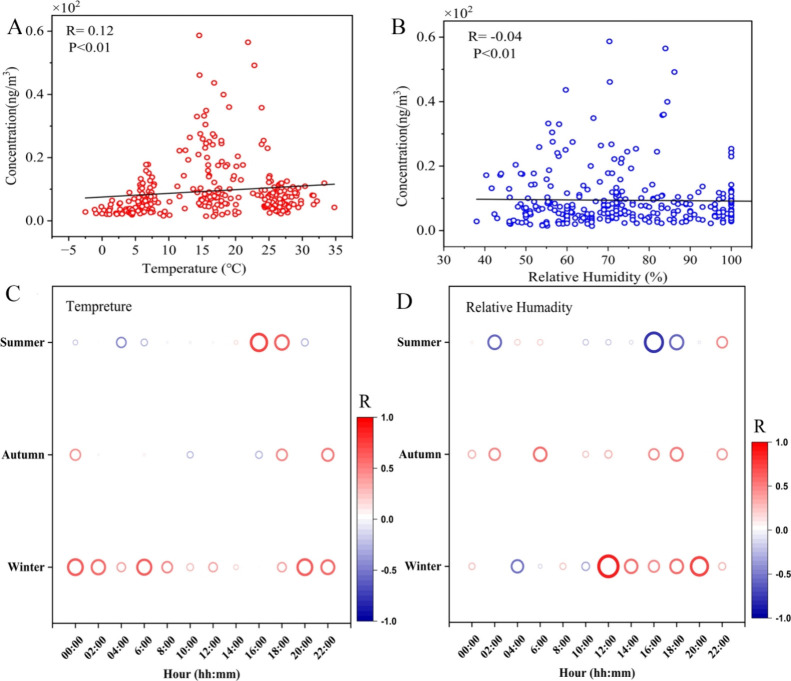
Scatter plot of TWCs concentrations against temperature (A) and relative humidity (B). Correlation (R) of TWCs concentrations with temperature (C), and relative humidity (D) at different times. *R* values in panels (A) and (B) indicate the correlation between fitted curves of TWC concentrations and temperature and between TWC concentrations and relative humidity, respectively.

In autumn, a relatively high positive correlation with temperature was observed from 21:00 to 00:00 at night and from 15:00 to 21:00 during the afternoon to evening peak hours. The consistent positive correlation with humidity throughout the time periods in autumn suggests that moisture accumulation on particles may enhance the retention of tire wear compounds. Higher temperatures contributed to increased tire wear particle production and exacerbated surface wear due to the rapid rise in tread temperature.^54,55^ A recent study by Kolomijeca et al. experimentally investigated how temperature altered the effects of leachates from tire particles on Fathead Minnow, showing a trend of increasing deformity severity with rising temperatures.^56^ However, under complex atmospheric conditions, these processes are likely to be influenced by a broader range of factors. These results deviate from our initial hypothesis, requiring further discussion and additional data.

When the data across different humidity intervals were examined (Figure S6), TWC concentrations peaked in the 80–90% RH range, reaching 11.64 ng/m^3^, followed by the 70–80% RH range at 10.63 ng/m^3^. In contrast, the concentrations of TWCs were significantly lower in the 30–40% RH interval (2.84 ng/m^3^), while those in the 60–70% RH interval showed a slight decline (7.73 ng/m^3^). This finding aligns with previous studies.^54^ Investigating the characteristics of tire wear particles under various nonvehicle operating conditions demonstrated that the quantity of fine wear particles (<10 μm) is significantly higher under water-lubricated conditions. Particularly, when surface roughness is low or under water-lubricated conditions, the wear mechanism is fatigue wear, which increases the likelihood of generating smaller particles. In contrast, at lower humidity levels, particle aggregation is reduced, leading to relatively lower concentrations of TWCs. Although tire wear compounds themselves are insoluble in water, changes in humidity can indirectly influence their concentration and distribution by affecting the physical and chemical properties of the particles. Tian et al. reported^52^ that concentrations of most TWCs positively correlated with RH (*R* = 0.327–0.794, *p* < 0.001–0.007), indicating that higher RHs and lower wind speeds reduce pollutant dispersion, thereby increasing the concentration of TWCs in ambient air.

In winter and summer, the correlation with humidity was higher during the evening rush hour and at night, suggesting that high nighttime concentrations may be driven by humidity. A previous study suggested that cold-climate regions may be particularly susceptible to contamination by tire-related chemicals due to the common use of winter tires and studded tires, poor road conditions from harsh climatic conditions, and the use of road salts, factors that collectively contribute to increased TWP production and enhanced additive leaching potential.

## Health Risk Implication

In recent years, various emerging organic pollutants with potential health risks have been identified in PM_2.5_, including halogenated polycyclic aromatic hydrocarbons and sulfur-containing polycyclic aromatic hydrocarbons.^57,58^ Due to the complex formation mechanisms and challenges in detection, these pollutants are often difficult to identify. Tire-related pollutants, particularly PPD antioxidants, have gained significant attention due to their widespread sources, such as the daily wear of rubber products such as automobile tires, personal safety belts, and electrical wire coatings.^38^ As a result, human exposure to these compounds is virtually unavoidable. Although the median concentrations of PM_2.5_-bound TWCs were in the pg/m^3^ range, repeated exposure through inhalation and ingestion raised concerns regarding human health. Given the significant variation in concentrations of these compounds in urban PM_2.5_, the health risks of inhalation exposure are gaining attention.

Studies indicate that the in vivo conversion rate of 6-PPD to 6-PPDQ does not exceed 2%; therefore, the potential health risks of 6-PPDQ are primarily linked to direct exposure via environmental media.^59^ In Tianjin, the detection rate of 6-PPDQ in the general population’s urine was significantly lower than in Shanghai and Guangzhou, with a lower median concentration.^39^ Zhang et al.^60^ estimated the daily intake (DI) and annual exposure dose (AED) of 6-PPDQ for adults based on its concentrations in particulate matter from six Chinese cities. The DI ranged from 2.2 pg to 1.3 ng/day, while the AED ranged from 0.5 ng to 0.4 μg/year. Dust ingestion has been identified as a major pathway for human exposure to 6-PPDQ, with traffic-related activities being a key factor influencing its environmental distribution. Zhang et al.^61^ reported that 6-PPDQ concentration in road dust from 55 cities across China was the highest in Changchun, exhibiting the highest concentration at 349 ng/g, highlighting the risk of human exposure. Estimated exposure levels via dust ingestion were 0.043 ng/(kg bw·day) for adults and higher for children at 0.076 ng/(kg bw·day). In high-exposure scenarios, both adult residents and children face increased intake levels. For instance, under medium-exposure scenarios, the estimated daily intake (EDI) for adults was 0.0159 ng/(kg bw·day), which rose to 0.0197 ng/(kg bw·day) in high-exposure scenarios.^35^

In our study, high concentrations of TWCs were observed at night. While previous studies indicated that the highest TWC concentration typically occurred during the evening rush hour,^19^ they also reported that the highest carcinogenic risks were observed at night, despite the reduced number of vehicles passing through the tunnel. Laboratory and on-road testing at the system level used real cars and chassis to assess specific variables under controlled test conditions such as different vehicle speeds and drive cycles on dynamometers. However, these tests were unable to represent the entire vehicle fleet or account for variations in driving styles. Additionally, climate and atmospheric processes in these controlled conditions did not reflect real-world emissions accurately, leading to discrepancies.^12^ Diurnal trend data can help individuals avoid high-exposure periods, thus reducing their daily inhalation of pollutants. The diurnal trends of TWCs in our study suggested that people could mitigate exposure by minimizing outdoor activities at night. Unfortunately, despite widespread environmental dispersal and potential human exposure to tire rubbers and elastomeric consumer products, public knowledge of their chemical composition remains limited. This is because their ingredients are typically protected as proprietary and confidential business information. Without regulatory requirements to disclose this information, environmental and human health risk assessments will remain challenging, uncertain, and prone to underestimation.

## Supplementary Material



## Notes

Supporting Information includes the sample extraction and processing (Text S1), information on chemical purchases, detailed estimates of physicochemical properties, and the MS parameters of TWCs used for HPLC–Q-TOF-MS/MS analysis (Tables S1–S5); reported concentrations of tire wear cyclic amines in air samples (Table S6), along with the PM_2.5_ concentration of sampling time period (Figure S1), and the molecular structures of target compounds and MS/MS fragment analysis (Figure S2); example of the identification of 8PPD and 44PD-Q (Figures S3 and S4); target MsMs fragment analysis of target TWCs (Figure S5); concentration of TWCs under different humidity levels (Figure S6) (PDF)
