# Supplementary material for: Tire Rubber-Derived Cyclic Amines in Urban Ambient Particulate Matter in Shanghai
Source: ACS Earth Space Chem. 2025 Mar 20;9(4):795–805. doi: 10.1021/acsearthspacechem.4c00291 (PMC12262421; doi:10.1021/acsearthspacechem.4c00291)
Supplement: Supplementary file 1 [file sp4c00291_si_001.pdf]

Tire Rubber-Derived Cyclic Amines in Urban Ambient Particulate Matter in Shanghai

Munila Abudumutailifu[†], Chengze Li[†], Haiping Xiong[†], Sihan Liu[†], Chunlin Li[‡], Dongmei Cai[†], Yinon Rudich[‖], Jianmin Chen[†,⁄⁄,⊥,*]

[†] Shanghai Key Laboratory of Atmospheric Particle Pollution and Prevention (LAP[3]), National Observations and Research Station for Wetland Ecosystems of the Yangtze Estuary, Department of Environmental Science & Engineering, Fudan University, Shanghai 200438, China.

[⊥] IRDR International Center of Excellence on Risk Interconnectivity and Governance on Weather/Climate Extremes Impact and Public Health, Institute of Atmospheric Sciences, Fudan University, Shanghai 200438, China.

[‡]College of Environmental Science and Engineering, Tongji University, Shanghai 200072, China

[‖]Department of Earth and Planetary Sciences, Weizmann Institute of Science, Rehovot 76100, Israel.

*Correspondence to: Jianmin Chen (jmchen@fudan.edu.cn)

Keywords: Tire wear chemicals; $PM_{2.5}$; Cyclic Amine; VACES; Health risks;

Text S1 Sample Extraction and Processing

To process each quartz filter, a specific procedure was followed. First, the filter punches were placed on ice and subjected to ultrasonic extraction. During this step, 10 mL of methanol was used to extract each filter punch over the course of 30 minutes, utilizing an Optima® LC/S ultrasonic bath (Fischer Scientific, USA). After the extraction, the collected extracts were passed through 0.22 μm polytetrafluoroethylene (PTFE) membranes to ensure filtration. These filtered extracts were then carefully moved into glass bottles that had been pre-cleaned and baked at 450°C for five hours to eliminate contaminants.

Next, the extracts were gently evaporated almost to dryness under a nitrogen (N_2) stream. Once reduced, the remaining residue was dissolved in 100 μL of methanol to ensure all target compounds were in solution. Following this, the extracts were cleaned up by spinning them in a centrifuge at 5000 rpm for 10 minutes. The resulting supernatant was then transferred into fresh tubes, ready for HPLC-Q-TOF-MS analysis. This meticulous process ensured that the samples were properly prepared and purified for the next analytical steps.

Table S1: Compound purchasing information including chemical supplier and commercial purity

Compound	CAS Number	Supplier	Purity
CHA	108-91-8	Macklin	>98%
DCHA	101-83-7	Macklin	>99.5%
DCA	7560-83-0	Macklin	>98%
Aniline	62-53-3	Aladdin	>99.5%
DPA	122-39-4	Macklin	>99%
DPG	102-06-7	Macklin	>97%
6PPD	793-24-8	Macklin	>98%
6PPD-Q	2754428-18-5	Toronto Research ChemicalsInc.	>98%
IPPD	101-72-4	Macklin	>98%
MBT	149-30-4	Macklin	>98%

Macklin Biochemical Technology Co., Ltd. (Shanghai, China)

Table S2. Detailed information on estimated physicochemical properties (log Kow, log Koa, vapor pressure, Henry's Law constant)(1)

Name	Log Kow[a] (estimated)	Log Koa[b] (estimated)	VPc (Pa)	Henry's Law constant
CHA	1.63	5.26	1.11×10^{3}	1.40
DCHA	3.30	7.02	3.38×10^{-2}	4.08×10^{-5}
DCA	3.71	6.88	4.13×10^{-2}	8.17×10^{-4}
Aniline	N/F*	N/F	N/F	N/F
DPA	3.50	7.64	6.70×10^{-4}	7.59×10^{-5}
DPG	2.89	12.4	5.29×10^{-4}	7.21×10^{-7}
6PPD	4.27	9.29	1.88×10^{-7}	7.69×10^{-8}
6PPD-Q	N/F*	N/F	N/F	N/F
IPPD	N/F*	N/F	N/F	N/F
MBT	N/F*	N/F	N/F	N/F

*N/F denotes that no property was reported;

Table S3. UHPLC-QTOF-MS method parameters

Instrument	HPLC-Q-Tof-Mass series 6540 (Agilent Technologies, Santa Clara, CA)		
Analytical column	ZORBAX Eclipse Plus C18 column (1.8 μm, 2.1 × 50 mm; Agilent Technologies)		
Injection Volume	2μl		
Mobile phase	A: Water+0.1% FA B: Methonal+0.1% FA		
Flow	0.35 mL min^{-1}		
LC setting	Time	A (%)	B (%)
	1	95	5
	3	75	25
	40	25	75
	65	0	100
	65.1	95	5
MS setting	Gas temp(℃)	300	
	Gas Flow (l/min)	8	
	Nebulizer (psig)	45	
	Sheath Gas Temp	350	
	Sheath Gas Flow	11	
	Polarity	Positive	
	Ion Source Type	ESI	
	Fragmentor	120	
	Skimmer1	65	
	MS range	50-1000	
MSMS setting	MS Scan Rate	5 spectra/s	
	MS/MS Scan Rate	4 spectra/s	
	Collision Energies	10V,20V,30V	

Table S4 Information on Suspect Tire Wear Compounds (TWCs)

Full Name	Abbreviations	Formula	Precursor Ion (m/z)	Confidence Level
N,*N*-Diisopropyl-*p*-phenylenediamine	33PD	$C_{12}H_{20}N_2$	193.1614	Level 2
N,*N′*-Di-sec-butyl-*p*-phenylenediamine	44PD	$C_{14}H_{24}N_2$	221.2045	Level 2
N,*N′*-Dicyclohexyl-*p*-phenylenediamine	CCPD	$C_{18}H_{28}N_2$	273.2383	Level 4
N-1-Methylheptyl-*N′*-phenyl-*p*-phenylenediamine	8PPD	$C_{20}H_{28}N_2$	297.2336	Level 2
N,N-Diisopropyl-p-phenylenediamine quinone	33PD-Q	$C_{12}H_{18}N_2O_2$	223.1435	Level 4
N,*N′*-Di-sec-butyl-*p*-phenylenediamine quinone	44PD-Q	$C_{14}H_{22}N_2O_2$	251.1749	Level 2
N,N'-Di(1-methylheptyl)-p-phenylenediamine quinone	88PD-Q	$C_{22}H_{38}N_2O_2$	363.3003	Level 2
N,*N′*-Dicyclohexyl-*p*-phenylenediamine quinone	CCPD-Q	$C_{18}H_{26}N_2O_2$	303.2065	Level 4
N-Isopropyl-*N′*-phenyl-*p*-phenylenediamine quinone	IPPD-Q	$C_{15}H_{16}N_2O_2$	257.1212	Level 4
N-(1,4-Dimethylpentyl)-*N′*-phenylbenzene-1,4-diamine quinone	7PPD-Q	$C_{19}H_{24}N_2O_2$	313.1937	Level 4
N-Decyl-*N*'-phenyl-p-phenylenediamine	$C_{22}H_{32}N_2$	$C_{22}H_{32}N_2$	325.2656	Level 4

Table S5: MS Parameters of TWCs used for HPLC–Q-TOF-MS/MS analysis.

Compound	Retention time /	Precursor Ion (m/z)	Product Ion (m/z) Top 4 Peaks	Collision Energy
CHA	1.19	100.1124	83.08;55.05;68.98;72.63;	10v
DCHA	4.90	182.1911	136.99;100.17;83.09;55.06;	10v
N-me-DCHA	4.712	196.2509	114.1308;83.0949;55.0610	20V
Aniline	0.73	94.0658	77.0386; 51.0230; 67.0541	20V
DPA	14.39	170.0966	152.06;93.06; 77.04;65.04;	40V
DPG	3.90	212.1814	195.09;119.06;94.06;77.04;	10v
IPPD	7.54	227.1540	184.09;107.06;93.05	20V
6PPD	12.46	269.2017	184.1031;107.0622;93.0605	30v
6PPD-Q	18.54	299.1775	256.11;241.09;215.08;187.08	20v
MBT	12.4/1	167.9936	124.02;109.01;133.07	20V

Table S6: Reported Concentrations of Tire Wear Cyclic Amines in Atmospheric aerosol Samples from Previous Studies

Name	Range(pg/m^3)	Location	References
DHCA	23-380	Swedish,	(2)
DHCA	4.1-82	Swedish	(3)
DPA	< 17	Swedish	(2)
DPG	45.0-199	global 19 megacities	(4)
DPG	0.10 – 97.5	Beijing	(5)
CHA	ND* – 4960	Beijing	(5)
aniline	224 ×10*3	Italy, Sticciano	(6)
aniline	7000-12×10*3	eastern Ontario	(7)
6PPD	22.2–6050	Guangzhou (Campus)	(8)
	2.23–9340	Guangzhou (Roadside)	
	1.02–3190	Taiyuan (Campus)	
6PPD	0.82–6.30	Hong Kong	(9)
6PPD	0.02−487	Taiyuan(Campus)	(10)
	1.2−109	Zhengzhou(Campus)	
	0.5−135	Shanghai(Campus)	
	0.4−75	Nanjing	
	0.1−6.0	Hangzhou	
	0.3−10	Guangzhou (Campus)	
6PPDQ	3.04–2350	Guangzhou (Campus)	(8)
	2.96–7250	Guangzhou (Roadside)	
	2.44–1780	Taiyuan (Campus)	
6PPDQ	0.54–13.8	Hong Kong	(9)
6PPDQ	ND*-1.75	Global 19 megacities	(4)
6PPDQ	1.1−84	Taiyuan(Campus)	(10)
	0.3−32	Zhengzhou(Campus)	
	0.3−39	Shanghai(Campus)	
	1.1−68	Nanjing	
	0.8−26	Hangzhou	
	0.1−15	Guangzhou (Campus)	

*ND means the compound was not detected.

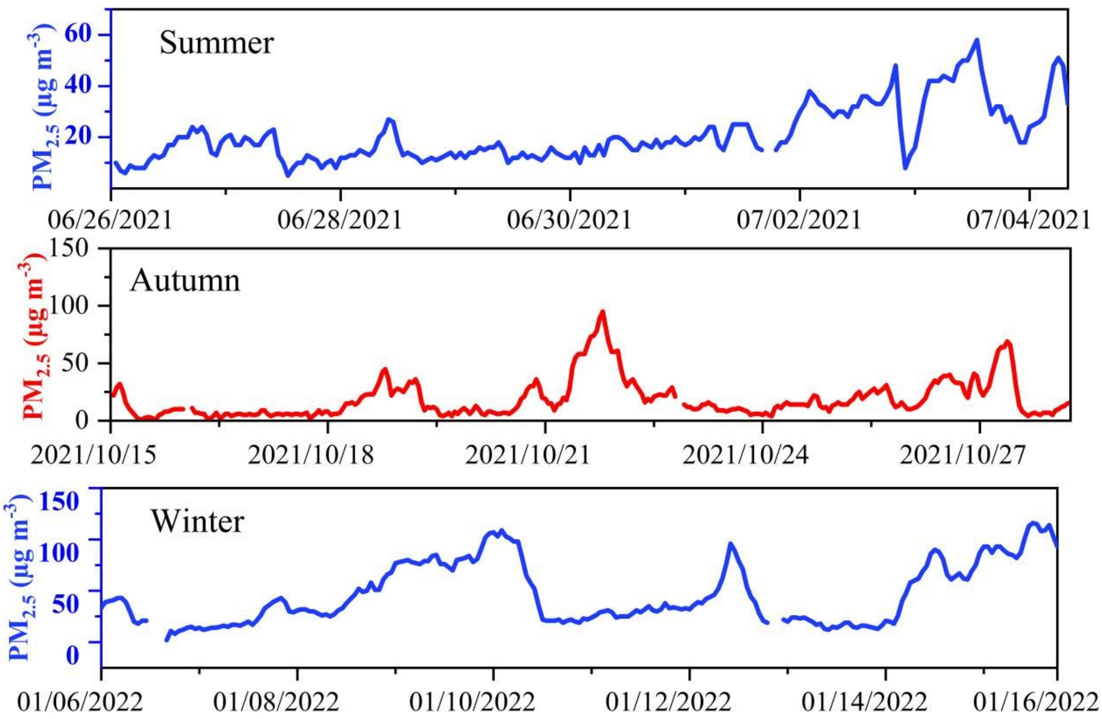

Figure S1. The $PM_{2.5}$ concentration of sampling time period

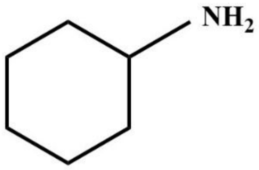

Cyclamine (CHA)

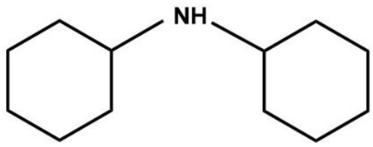

Dicyclohexylamine (DCHA))

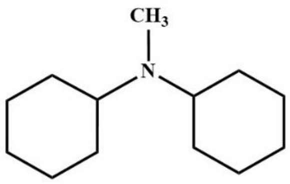

1-cyclohexyl-3-phenylurea (CPU))

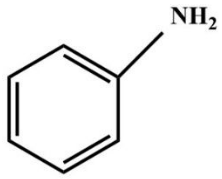

Aniline (ANI))

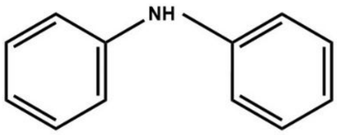

diphenylamine (DPA))

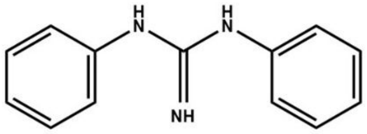

1,3-diphenylguanidine (CPG)

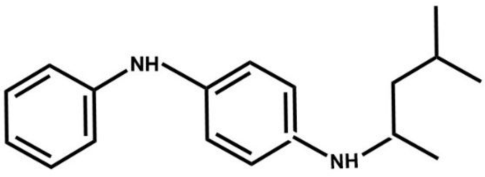

N-phenyl-N′ -(1, 3-dimethylbutyl)-p-phenylenediamine (6PPD)

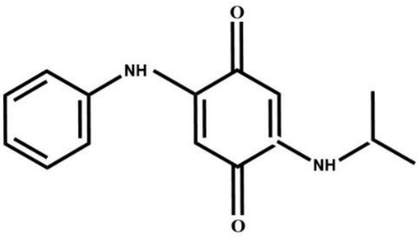

N-(1,3-dimethylbutyl)-N′-phenyl-p-phenylenediamine quinone (6PPD-Q)

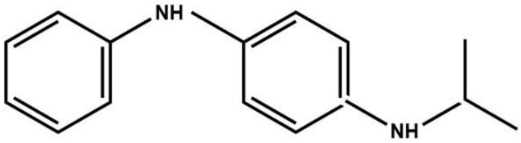

4-(Isopropamino)diphenamine (IPPD)

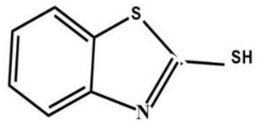

Mercaptobenzothiazole (MBT)

Figure S2. Molecular structures of target TWCs in this study.

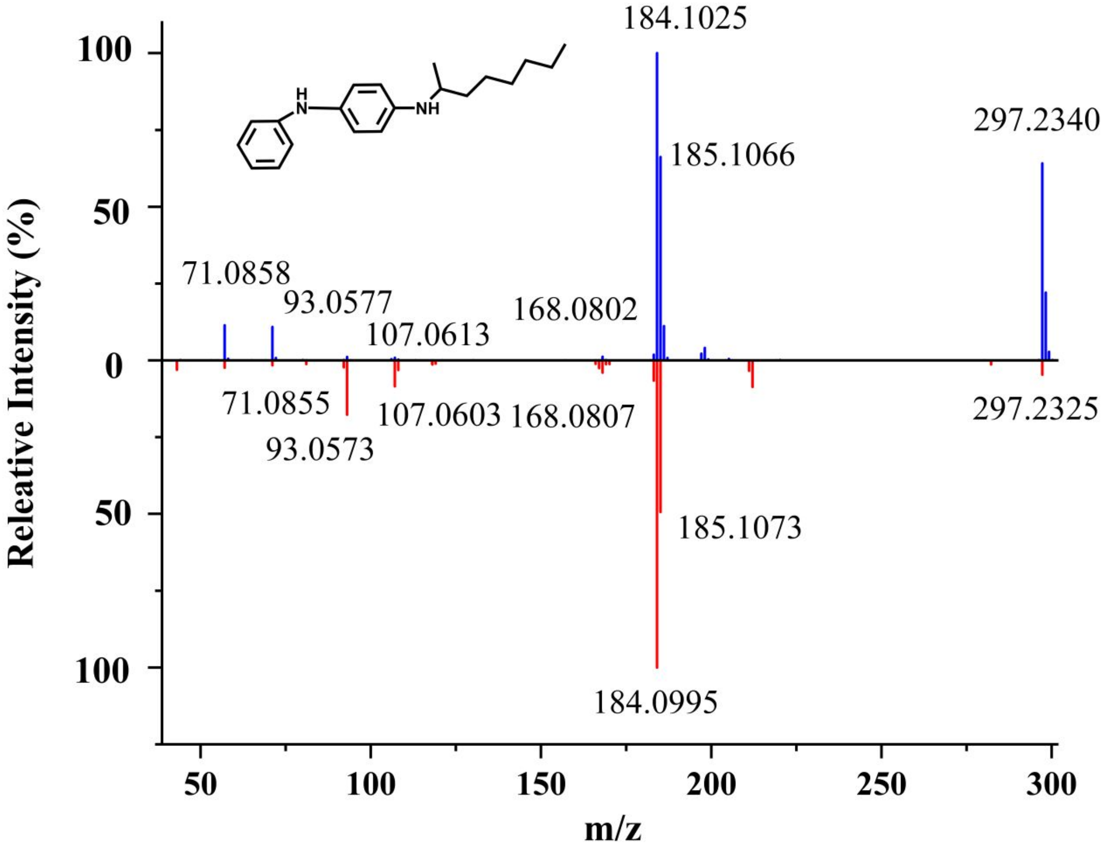

Figure S3 Example of the identification of the compound 8PPD using MS/MS spectra and corresponding fragments of Mass Bank database. The red color represents MassBank fragments, while the blue color represents sample fragments.

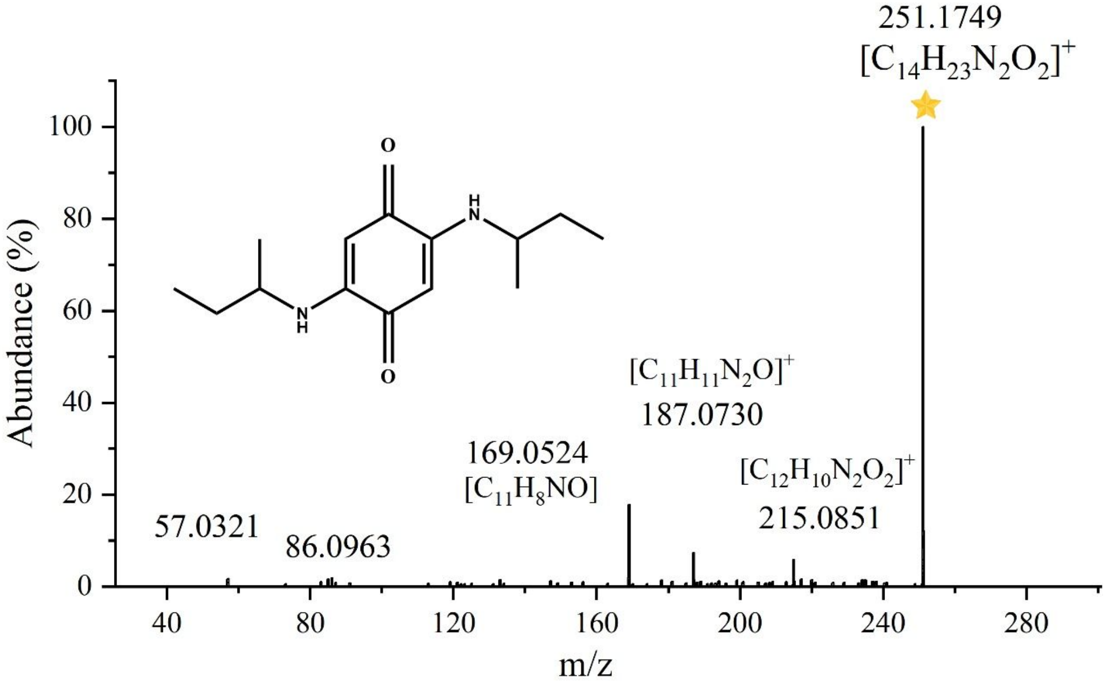

Figure S4 Example of the identification of the compound 44PD-Q using MS/MS spectra and corresponding fragments.

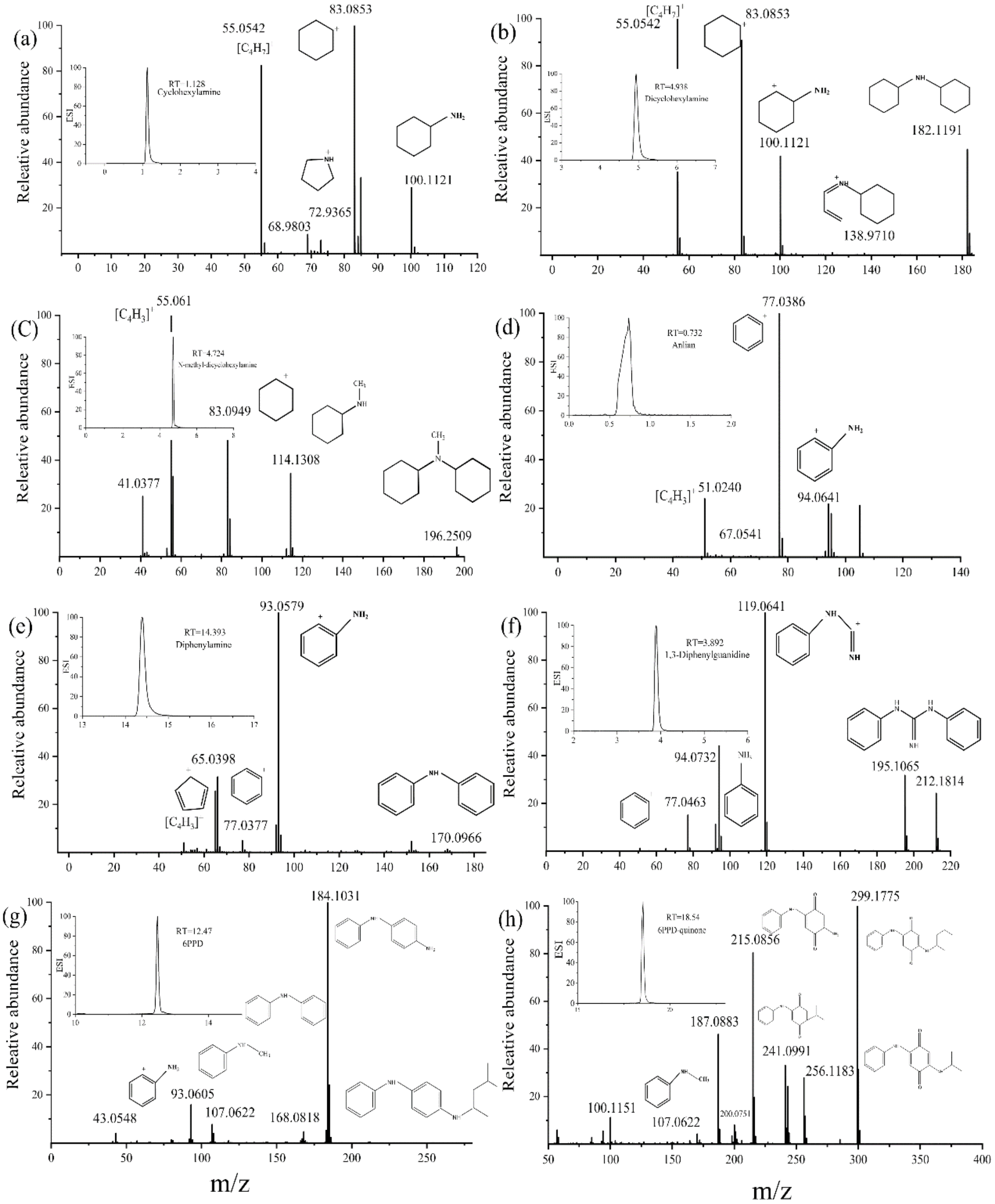

Figure S5. The MsMs Fragment analysis of Target TWCs.

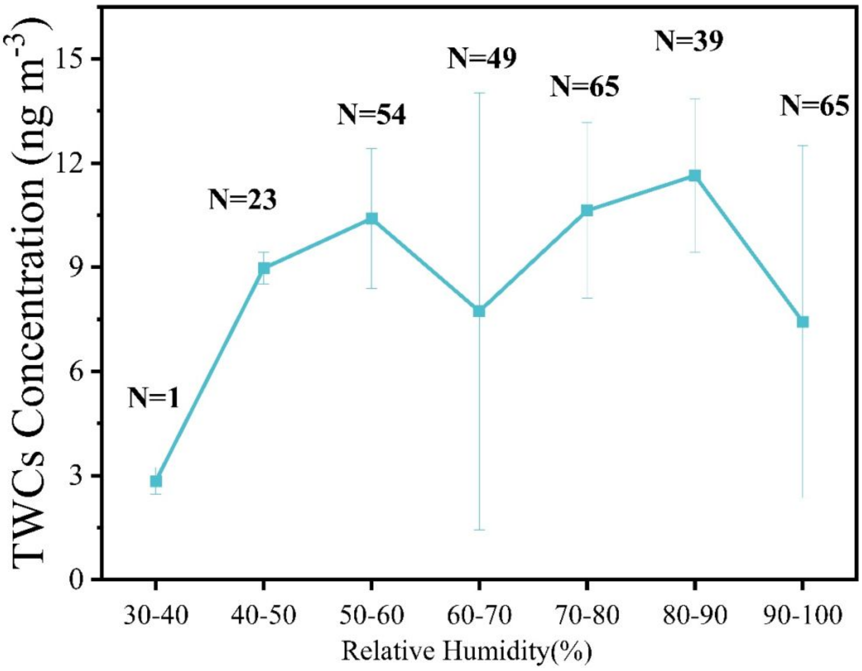

Figure S6 Concentration of TWCs under Different Humidity Levels

References

1. Johannessen, C.; Liggio, J.; Zhang, X.; Saini, A.; Harner, T. Composition and transformation chemistry of tire-wear derived organic chemicals and implications for air pollution. *Atmos. Pollut. Res.* **2022,** *13*, 101533.

2. Woldegiorgis, A.; Wiklund, P.; Remberger, M.; Kaj, L.; Viktor, T.; Lilja, K.; Brorström-Lundén, E. Results from the Swedish National Screening Programme 2007. *IVL Swedish Environmental Research Institute Ltd.* **2008**.

3. Eva Brorström-Lundén; Katarina Hansson; Mikael Remberger; Lennart Kaj; Jörgen Magnér; Hanna Andersson, I. Screening of benzothiazoles, benzenediamines, dicyclohexylamine and benzotriazoles. *Swedish Environmental Protection Agency.* **2011**.

4. Johannessen, C.; Saini, A.; Zhang, X.; Harner, T. Air monitoring of tire-derived chemicals in global megacities using passive samplers. *Environ Pollut.* **2022,** *314*, 120206.

5. Lin, Y.; Yang, J.; Fu, Q.; Ruan, T.; Jiang, G. Exploring the Occurrence and Temporal Variation of ToxCast Chemicals in Fine Particulate Matter Using Suspect Screening Strategy. *Environ. Sci. Technol.* **2019,** *53*, 5687-5696.

6. Palmiotto, G.; Pieraccini, G.; Moneti, G.; Dolara, P. Determination of the levels of aromatic amines in indoor and outdoor air in Italy. *Chemosphere.* **2001,** *43*, 355-361.

7. Zhu, J.; Aikawa, B. Determination of aniline and related mono-aromatic amines in indoor air in selected Canadian residences by a modified thermal desorption GC/MS method. *Environment International.* **2004,** *30*, 135-143.

8. Wang, W.; Cao, G.; Zhang, J.; Wu, P.; Chen, Y.; Chen, Z.; Qi, Z.; Li, R.; Dong, C.; Cai, Z. Beyond Substituted p-Phenylenediamine Antioxidants: Prevalence of Their Quinone Derivatives in PM2.5. *Environ. Sci. Technol.* **2022,** *56*, 10629-10637.

9. Cao, G.; Wang, W.; Zhang, J.; Wu, P.; Zhao, X.; Yang, Z.; Hu, D.; Cai, Z. New Evidence of Rubber-Derived Quinones in Water, Air, and Soil. *Environ. Sci. Technol.* **2022,** *56*, 4142-4150.

10. Zhang, Y.; Xu, C.; Zhang, W.; Qi, Z.; Song, Y.; Zhu, L.; Dong, C.; Chen, J.; Cai, Z. p-Phenylenediamine Antioxidants in PM2.5: The Underestimated Urban Air Pollutants. *Environ. Sci. Technol.* **2021,** *56*, 6914-6921.
